# Traumatic Experiences, Stressful Events, and Alexithymia in Chronic Migraine With Medication Overuse

**DOI:** 10.3389/fpsyg.2018.00704

**Published:** 2018-05-14

**Authors:** Sara Bottiroli, Federica Galli, Michele Viana, Grazia Sances, Cristina Tassorelli

**Affiliations:** ^1^Headache Science Centre, IRCCS Mondino Foundation, Pavia, Italy; ^2^Department of Health Sciences, University of Milan, Milan, Italy; ^3^Department of Brain and Behavioral Sciences, University of Pavia, Pavia, Italy

**Keywords:** trauma, stress, alexithymia, medication overuse headache, migraine

## Abstract

**Background:** Many factors are involved in the prognosis and outcome of Chronic Migraine and Medication Overuse Headache (CM+MOH), and their understanding is a topic of interest. It is well known that CM+MOH patients experience increased psychiatric comorbidity, such as anxiety, depression, or personality disorders. Other psychological factors still need to be explored. The present study is aimed to evaluate whether early life traumatic experiences, stressful life events, and alexithymia can be associated with CM+MOH.

**Methods:** Three hundred and thirty-one individuals were recruited for this study. They belonged to one of the two following groups: CM+MOH (*N* = 179; 79% females, Age: 45.2 ± 9.8) and episodic migraine (EM) (*N* = 152; 81% females; Age: 40.7 ± 11.0). Diagnosis was operationally defined according to the International Classification of Headache Disorders 3rd edition (ICHD-IIIβ). Data on early life (physical and emotional) traumatic experiences, recent stressful events and alexithymia were collected by means of the Childhood Trauma Questionnaire, the Stressful life-events Questionnaire, and the Toronto Alexithymia Scale (TAS-20), respectively.

**Results:** Data showed a higher prevalence of emotional (χ^2^ = 6.99; *d.f*. = 1; *p* = 0.006) and physical (χ^2^ = 6.18; *d.f*. = 1; *p* = 0.009) childhood trauma and of current stressful events of important impact (χ^2^ = 4.42; *d.f*. = 1; *p* = 0.025) in CM+MOH patients than in EM ones. CM+MOH patients were characterized by higher difficulties in a specific alexithymic trait (Factor 1 subscale of TAS-20) [*F*_(1, 326)_ = 6.76, *p* = 0.01, η_*p*_^2^ = 0.02] when compared to the EM group. The role of these factors was confirmed in a multivariate analysis, which showed an association of CM+MOH with emotional (OR 2.655; 95% CI 1.153–6.115, *p* = 0.022) or physical trauma (OR 2.763; 95% CI 1.322–5.771, *p* = 0.007), and a high score at the Factor 1 (OR 1.039; 95% CI 1.002–1.078, *p* = 0.040).

**Conclusions:** Our findings demonstrated a clear relationship between CM+MOH and life traumas, stressful events, and alexithymia. These observations have a relevant role in multiple fields of related to chronic headache: from the management to the nosographic framing.

## Introduction

Childhood trauma refers to psychological, physical, emotional or sexual abuse, and emotional neglect occurring before age 17 years (Bernstein et al., 2003). Stressful life events are instead defined as those recent events that could determine a substantial change in one's positive or negative personal circumstances (Brown and Birley, 1968). Both childhood trauma and stressful events seem to contribute in the development of chronic pain syndromes (Lampe et al., 2003). This because they affect nervous, immune, and endocrine systems and are associated to the onset and perpetuation of pain later in adulthood (Lampe et al., 2003). In this field, headache deserves a special mention being one of the most common and disabling disorders of the nervous system. In most sufferers, attacks recur episodically, however a small, but significant, portion of patients evolve into chronic migraine, often occurring in association an excessive use of acute medications (medication overuse headache – CM+MOH) (Olesen et al., 2006), such as combination of analgesics, triptans, or non-steroidal anti-inflammatory drugs (Da Silva and Lake, 2014). CM+MOH is strongly associated with psychiatric conditions (mainly anxiety and depression) or personality disorders (Sances et al., 2010, 2013; Bottiroli et al., 2016). Since 1990 (Merikangas et al., 1990), the association of psychiatric disorders and headache has been labeled as comorbidity. Defining the factors that are involved in the association headache with psychiatric comorbidity is compelling and helpful to overcome the *cul de sac* of a long-lasting inexplicable association (Galli, 2017).

For instance, the association among early traumatic experiences, stressful events, and the development and perpetuation of chronic pain syndromes (Afari et al., 2014) may be due, according to the biopsychosocial model, to a complex interaction among biological, psychological, and psychosocial factors reciprocally influencing each other (Andrasik et al., 2005). We know that childhood trauma (Peterlin et al., 2007; Fuh et al., 2010; Tietjen et al., 2010; Tietjen, 2016) and the exposure to stressful events (D'Amico et al., 2000; Scher et al., 2003; Schramm et al., 2015) are able to increase headache frequency, severity, and chronicity of different headache disorders. In particular, headache in people with childhood abuse seems to be more disabling and frequent and more likely to transform from episodic to chronic (Tietjen et al., 2010). Self-reported early life traumas are, indeed, more prevalent in CM patients when compared with those with episodic migraine (Peterlin et al., 2007). Similarly, stressful life events are among risk factors for the development of chronic migraine (Lipton, 2009). Moreover, people being victim of abuse in childhood are also those being more likely re-victimized across life span, being exposed to increased stressful life events (Tietjen et al., 2010). However, only a limited number of studies has explored this topic in CM+MOH as a separate group among chronic headache sub-types. In this field, a Turkish study (Altintaş et al., 2015a) failed to detect differences in the occurrence of early life maltreatments among CM+MOH, chronic, and episodic migraine. We recently found that childhood trauma, together with history of depression, are predictors of medication overuse headache (Viana et al., 2018). As for current stressful events, a study by the Danish group (Westergaard et al., 2016) reported a strong link between CM+MOH and stress, even if another Turkish research failed to identify such a relationship between medication overuse and number of events (Altintaş et al., 2015b). Thus, more research is needed in order to shed light on this argument.

Alexithymia refers to the difficulty in term of emotional awareness, which is the ability to identify and recognize emotions in oneself and in the others (Taylor, 2001). An important current discussion in this field concerns whether alexithymia could be considered as a mental state or a stable personality trait (Taylor et al., 1997). A good position considers that alexithymia in subjects with substance use disorders who underwent detoxification is both a state and trait phenomenon and does not appear to be related to changes in anxiety- and depression-like symptoms (de Haan et al., 2014). Alexithymia is common in chronic pain syndromes (Gulec et al., 2004; Sayar et al., 2004), such as chronic headache associated or not with medication overuse (Galli et al., 2017). Other fields of research have shown the existence of an association between alexithymia and childhood trauma (Frewen et al., 2008) and stressful life events (Honkalampi et al., 2004). A limited number of studies explored the occurrence of alexithymia in migraine (Balaban et al., 2012; Karşikaya et al., 2013; Galli et al., 2017).

Our hypothesis is that childhood trauma, adult stressful life events (stressful events within the last 10 years) and alexithymia interact with one another to influence the development of CM+MOH. In the present study, we therefore aimed at evaluating whether early life traumatic experiences, stressful life events, and alexithymia are associated—on both an individual and group level—with CM+MOH. Their presence in a subject with migraine would therefore define a subpopulation of patients who are at a high risk of a negative outcomes.

## Materials and methods

### Subjects

This study was conducted at the Pavia Headache Center (a tertiary referral center) of the Mondino Foundation in Pavia, Italy. We enrolled consecutive patients with long-term episodic migraine (EM, history of illness >10 years who never developed MOH) and patients with CM+MOH. The study was approved by the Ethics Committee of San Raffaele Scientific Institute (Milan, Italy) and written informed consent was obtained from all patients. Recruitment started on March 2014 and was completed in July 2016. All consecutive EM and CM+MOH patients were enrolled. EM patients were enrolled in headache clinics; CM+MOH patients were enrolled during the inpatient detoxification program.

#### EM patients

Inclusion criteria for patients with EM were: (a) age >18, < 65 years, (b) fulfillment of ICHD-III beta criteria for migraine with or without aura, (c) migraine duration >10 years. Exclusion criteria were: (a) previous or present history of MOH or any other type of chronic headache (ICHD-III beta), (b) dementia, (c) previous diagnosis of psychosis, and (d) mental retardation. Verification of the eligibility criteria was made by an expert neurologist.

#### CM+MOH patients

Inclusion criteria for patients with CM+MOH were: (a) age >18, < 65 years, (b) fulfillment of ICHD-III beta criteria for CM and MOH. Exclusion criteria were: (a) dementia, (b) previous diagnosis of psychosis, and (c) mental retardation. Verification of the eligibility criteria was made by an expert neurologist.

### Procedures

Each consultation was performed by a neurologist that diagnosed the headache type, collected socio-demographic data, migraine characteristics and history, and history of present and previous use of medications and/or other substances. Participants also filled a series of self-report questionnaires.

CM+MOH patients underwent a complete evaluation based on the DSM criteria by an expert psychologist (SB or FG). EM patients were screened for anxiety and depression using the Hospital Anxiety and Depression Scale (Zigmond and Snaith, 1983).

For the detection and characterization of trauma, we used a shorter Italian version (adapted by Bottiroli et al., 2017) of the Childhood Trauma Questionnaire (Bernstein et al., 2003), which includes 13 items referring to different types of childhood trauma such as emotional abuse, emotional neglect, physical abuse, physical neglect, and sexual abuse. This version of the scale, as evaluated in the present study, has moderate internal consistency (Cronbach's alpha = 0.51). For each item, patients were requested to indicate whether or not they experienced each kind of trauma. We considered the number of participants reporting traumatic experiences, by considering separately physical and emotional trauma, also in terms of neglect and abuse. Stressful life-events were assessed via the questionnaire proposed by Paykel et al. (1971), subsequently, adapted to the Italian language by Bottiroli et al. (2017). The questionnaires consists in a list of 58 stressful life events (e.g., moving, divorce, new work, dismissal, etc.), and has a good internal consistency (Cronbach's alpha = 0.73), as resulted from our data. Patients were requested to tick those events that occurred to them in the last 10 years. We considered the total number of participants reporting stressful events. Participants were also requested to rate the impact of each of the selected events on their quality of life, on a 5-points Likert scale ranging from mild to very serious. We also counted the number of participants ticking each level of impact. A further index derived from considering the number of participants reporting events with at least an “important” level of impact, which derived by summing the important, serious, and very serious impacts. In order to understand the additional burden of having experienced both childhood trauma and current stress, we also considered the number of participants reporting both types of event.

The presence of alexithymia was investigated using the 20-item Italian version (Caretti et al., 2005) of the Toronto Alexithymia Scale (TAS-20; Bagby et al., 1994), which has a good internal consistency (Cronbach's alpha = 0.81) as based on literature, and contains the same three-factor structure as the English version of the scale. Items in the first factor (Factor 1) are referred to the ability to identify feelings and distinguish them from bodily sensations. Items in the second factor (Factor 2) relate to a concrete thinking style. Items in the third factor (Factor 3) concern the ability to express emotion and fantasy (daydreaming). The total scores for the TAS-20 were categorized as follow: a score >61 indicated the presence of alexithymia, a score of < 51 indicated the absence of alexithymia; a score between >51 and < 61 indicated a borderline level (Caretti et al., 2005).

### Statistical procedures

Data are presented as means ± *SD* for continuous data and as n/% for frequency data. The differences between EM and CM+MOH were examined with χ^2^ tests for categorical variables and one-way analysis of variance (ANOVA) for quantitative variables. Univariate and multivariate logistic regression (enter method) were applied. The criterion for variables inclusion in univariate model was the existence of significant differences among groups in the ANOVAs, whereas for variables inclusion in multivariate model was statistical significance at the level of *p* ≤ 0.05, obtained by univariate analysis. An alpha of 0.05 was used for all statistical tests. All analyses were conducted using SPSS (Statistical Package for Social Sciences, version 23.0).

## Results

### Patient population

Three hundred and thirty-one patients were enrolled, of which 152 suffered from EM and 179 from CM+MOH. All CM+MOH patients enrolled had a previous multiannual history of EM.

#### EM patients

Patients had the following characteristics: 81% were female (*n* = 123), average age was 40.7 ± 11.0, the average age at onset of migraine was 16.3 ± 8.7. The average monthly frequency of migraine attacks was 6.4 ± 3.3.

#### CM+MOH patients

Patients had the following characteristics: 79% were female (*n* = 141), average age was 45.2 ± 9.8, the average age at onset of migraine was 14.3 ± 8.4, the average age at onset of CM+MOH was 23.8 ± 5.5.

The average frequency of headache days per month was 26.1 ± 4.8, the average frequency of days of medication intake per month was 23.8 ± 5.5, and the average frequency of medication use per month was 45.6 ± 34.7. No patients had abuse of opioids.

### Comparison between EM and CM+MOH patients

When comparing demographic and clinical features (Table 1) between these 2 groups, we observed a older age [*F*_(1, 319)_ = 15.12, *p* < 0.001], lower age at onset of migraine [*F*_(1, 317)_ = 4.57, *p* = 0.033], and higher frequency of attacks per months [*F*_(1, 190)_ = 1073.82, *p* < 0.001] in CM+MOH patients when compared to EM patients.

**Table 1:** Demographic, clinical, and psychological characteristics of medication overuse headache (CM+MOH) and episodic migraine (EM) patients.

	**CM+MOH *N* = 179 mean ± *SD***	**EM *N* = 152 mean ± *SD***	***p***
Age	**45.2** ± **9.8**	**40.7** ± **11.0**	<**0.001**
Gender (Female) *n* (%)	141 (79%)	123 (81%)	0.37
Age at onset	**14.3** ± **8.4**	**16.3** ± **8.7**	**0.033**
Drug doses per month	45.6 ± 34.7	–	–
Days with headache per month	**26.1** ± **4.8**	**6.4** ± **3.3**	<**0.001**
**CHILDHOOD TRAUMA QUESTIONNAIRE** ***n*** **(%)**			
Traumatic experiences (yes)	**111 (62%)**	**73 (48%)**	**0.007**
Emotional trauma (yes)	**87 (49%)**	**52 (34%)**	**0.006**
Emotional abuse (yes)	20 (11%)	11 (7%)	0.19
Emotional neglect (yes)	**84 (47%)**	**51 (34%)**	**0.005**
Physical trauma (yes)	**65 (37%)**	**36 (24%)**	**0.009**
Physical abuse (yes)	**26 (15%)**	**12 (8%)**	**0.05**
Physical neglect (yes)	**54 (30%)**	**31 (20%)**	**0.023**
**STRESSFUL LIFE-EVENTS QUESTIONNAIRE** ***n*** **(%)**			
Life events (yes)	172 (96%)	132 (92%)	0.075
Mild events (yes)	67 (38%)	63 (45%)	0.13
Moderate events (yes)	104 (59%)	84 (57%)	0.49
Important events (yes)	133 (75%)	96 (68%)	0.087
Very serious events (yes)	41 (23%)	29 (20%)	0.30
Serious events (yes)	77 (43%)	52 (36%)	0.12
At least important events (yes)	**151 (85%)**	**108 (76%)**	**0.025**
N. of childhood trauma and stressful life events combined (%) (yes)	**107 (60%)**	**67 (47%)**	**0.012**
**TAS-20 (TORONTO ALEXITHYMIA SCALE)**			
Alexithymic patients *n* (%)	58 (33%)	44 (29%)	0.30
Total score (20–100)	46.1 ± 11.7	44.4 ± 12.4	0.20
Factor 1 (7–35)	**16.1** ± **6.7**	**14.3** ± **6.3**	**0.01**
Factor 2 (5–25)	12.3 ± 4.7	12.4 ± 4.7	0.89
Factor 3 (8–40)	17.7 ± 4.6	17.7 ± 4.7	0.92

*Possible range scores are indicated enclosed by brackets. Significant differences are visualized in bold*.

As regards psychological variables, as measured via self-report questionnaires, data showed a (Table 1) significant association between group and childhood trauma in CM+MOH patients as compared to EM ones. The higher prevalence of childhood trauma (χ^2^ = 6.51; *d.f*. = 1; *p* = 0.007) in CM+MOH involved both emotional (χ^2^ = 6.99; *d.f*. = 1; *p* = 0.006) and physical (χ^2^ = 6.18; *d.f*. = 1; *p* = 0.009) trauma. An higher prevalence in term of emotional neglect (χ^2^ = 8.02; *d.f*. = 1; *p* = 0.005), physical neglect (χ^2^ = 5. 19; *d.f*. = 1; *p* = 0.023), and physical abuse (χ^2^ = 4.14; *d.f*. = 1; *p* = 0.05) was also found in CM+MOH patients as compared to EM ones.

As for stressful life events, we found a significant association between group and stressors of at least important impact, with a higher prevalence of them (χ^2^ = 4.42; *d.f*. = 1; *p* = 0.025) in CM+MOH patients than in EM ones. No other differences resulted across groups as concerns the stressful life-events of minor impact (*p*s > 0.075).

We also detected a significant association between group and the presence of both childhood trauma and current stressful events, with a higher prevalence of this combination of adversities in CM+MOH patients (χ^2^ = 5.64; *d.f*. = 1; *p* = 0.018) than in EM ones.

As for TAS-20, CM+MOH patients were characterized by higher Factor 1 scores [*F*_(1, 326)_ = 6.76, *p* = 0.01, η_*p*_^2^ = 0.02] when compared to the EM group.

No other significant differences resulted between these two groups of patients.

### Predictors of medication overuse headache

According to the results of a univariate analysis (Table 2), the factors associated to the presence of medication overuse headache were: presence of emotional (OR 1.819; 95% CI 1.165–2.838, *p* = 0.022) or physical trauma (OR 1.837; 95% CI 1.134–2.976, *p* = 0.013), presence of at least important current stressful life events (OR 1.828; 95% CI 1.037–3.224, *p* = 0.037), presence of both childhood trauma and stressful life events (OR 1.708; 95% CI 1.096–2.661, *p* = 0.018), and a high score at the Factor 1 subscale of TAS-20 (OR 1.046; 95% CI 1.010–1.082, *p* = 0.010). The presence in general of childhood trauma, as well as of emotional and physical abuse/neglect were not introduced in the model given that we separately considered emotional and physical trauma.

**Table 2:** Model fit of logistic regression equations to predict chronic migraine and medication overuse headache (CM+MOH).

	**Univariate OR**	**95% CI**	***p*-value**	**Multivariate OR**	**95% CI**	***p*-value**
**CHILDHOOD TRAUMA QUESTIONNAIRE**						
Emotional trauma (yes vs. no)	**1.819**	**1.165–2.838**	**0.008**	**2.655**	**1.153–6.115**	**0.022**
Physical trauma (yes vs. no)	**1.837**	**1.134–2.976**	**0.013**	**2.763**	**1.322–5.771**	**0.007**
**STRESSFUL LIFE EVENTS QUESTIONNAIRE**						
At least important events (yes vs. no)	**1.828**	**1.037–3.224**	**0.037**	–	–	0.18
No. of childhood trauma and stressful life events combined (yes vs. no)	**1.708**	**1.096–2.661**	**0.018**	–	–	0.12
**TAS-20 (TORONTO ALEXITHYMIA SCALE)**						
Factor 1	**1.046**	**1.010–1.082**	**0.010**	**1.039**	**1.002–1.078**	**0.04**

*Significant ORs are in indicated bold*.

In a multivariate analysis, the factors that emerged as predictor of medication overuse headache were: the presence of emotional (OR 2.655; 95% CI 1.153–6.115, *p* = 0.022) or physical trauma (OR 2.763; 95% CI 1.322–5.771, *p* = 0.007), and a high score at the Factor 1 subscale of TAS-20 (OR 1.039; 95% CI 1.002–1.078, *p* = 0.040). This logistic regression model was statistically significant, $\chi_{\left(5\right)}^{2}$ = 22.27, *p* < 0.001 and it explained 9.1% (Nagelkerke *R*^2^) of the variance of medication overuse headache and correctly classified 63.3% of cases.

## Discussion

The results of the present study showed that history of childhood (emotional and physical) trauma together with stressful events of important impact, and difficulties to identify feelings and distinguish them from bodily sensation are substantial determinants of CM+MOH. By contrast, CM+MOH and EM patients are comparable in terms of stressful events of minor impact as well as in the other alexithymic traits. Noteworthy, the prevalence of early traumatic experiences (both physical and emotional) in the group with EM is impressive (48%), and higher than estimates from epidemiological studies in under 13-year old population (38%) (Koenen et al., 2010). The very fact that the sum of childhood trauma and stressful life events is significantly higher in CM+MOH than EM evidences a trend toward a matched growing of headache severity and psychological vulnerability. This is further underlined by the marked alexithymic profile showed by CM+MOH as well. Studies of nonclinical populations also have found that childhood abuse predicts deficits in emotional awareness and expression (Paivio and McCulloch, 2004). From another side, both clinical and community studies indicated that individuals with chronic pain were also more likely to report a history of abuse or neglect compared to those who reported less pain symptoms or conditions (Davis et al., 2005).

In the present study, we focused on a specific type of chronic pain, which is complicated by medication overuse. In other research areas, it has been shown that early life adversities represent risk factors increasing the likelihood to develop a variety of psychiatric disorders, including substance abuse and dependence behaviors (Kendler et al., 2000; Dube et al., 2003; Widom et al., 2006), due to psychological and physiological changes induced by early and prolonged exposure to stress (Cohen et al., 1995). The question of whether overuse of pain-relieving drugs is the cause or the consequence of the increased headache frequency is still a matter for debate. Previous evidence puts CM+MOH and dependence together, suggesting a common underlying vulnerability (Radat et al., 2008), partly due to psychiatric comorbidity (Limmroth et al., 2002; Saper et al., 2005). Others argued that substance abuse behaviors in CM+MOH varied according to the types of symptomatic drugs overused (Fuh et al., 2005; Fumal et al., 2006). By contrast, our group (Galli et al., 2011) has previously shown that MOH and drug addicted patients differed in those personality aspects related to dependence and that probably CM+MOH patients overuse medications because they need to cope with life in spite of daily attacks of headache. A likely explanation is that the exposure to childhood traumas causes a difficulty in these patients to tolerate recurrent pain, making them particularly prone to medication misuse. This point is supported by previous evidence (Teicher and Samson, 2013) showing that individuals having experienced childhood trauma represent “ecophenotypic variants as clinically and neurobiologically distinct subtypes” (p. 1114) and they differ from non-maltreated persons in how they react to the same conditions. Given that we did not include a group of chronic migraineurs without overuse, our results are only speculative and need to be confirmed by exploring how these patients respond to detoxification treatments.

This study advances our knowledge of trauma in CM+MOH by exploring the impact of emotional and physical events, both resulting more prevalent in this population of patients and resulting as significant antecedents of more complicated expressions of migraine. What is interesting here is the investigation of emotional trauma, which represents a less recognized and explored type of maltreatment with respect to physical and sexual abuse (Edwards et al., 2003). Other studies before us have shown how emotional and physical trauma may have consequences on migraine characteristics (Tietjen et al., 2010; Kucukgoncu et al., 2014; Demiryürek et al., 2017). Interestingly, we found that irrespective of the type of trauma, any form of early life adversity may impact this condition, probably representing a kind of epigenetic cue with still unknown influences on cerebral mechanisms related to pain. It is unknown whether the influence of traumatic experience is related specifically to migraine or to chronic pain in general, and in this case why a traumatized patient develops migraine and no other types of pain.

Stress is well known as one of the most common headache trigger factors, which can increase headache frequency and promote headache chronification (Mosley et al., 1991). CM+MOH patients in our study were characterized by a higher number of “at least important” stressful events than episodic controls. It would be interesting to understand whether CM+MOH patients are exposed to a greater number of stressful live events or they are less able to tolerate and cope with stress than episodic migraineurs. In literature there is limited evidence showing a greater presence of stressors in chronic headache patients than in healthy controls (Invernizzi et al., 1985; De Benedictis and Lorenzetti, 1992). By contrast, stress derives from the inability to deal with experienced life events (Nash and Thebarge, 2006) and chronic headache patients tend to be less able to adopt coping strategies than healthy controls (Dube et al., 2003; Widom et al., 2006). In this frame, our findings support the hypothesis of a greater psychological vulnerability in the development of chronic headache associated to acute medication overuse. Future studies should explore such findings according to the duration of the exposure to these stressors and/or the mediator effect of other variables (e.g., personality or cognitive characteristics).

A specific alexithymic trait—difficulty in identifying feeling—distinguished medication overuse headache patients from episodic migraineurs. This result corroborates the idea that alexithymia could represent a risk factor increasing susceptibility to disease (Weiner, 1982). This is not particularly surprising given that this tendency has been previously highlighted in chronic migraineurs with medication overuse (Galli et al., 2017). What is new here is the fact that we extended these results in the frame of coexisting childhood traumas and life events. According to etiological studies, alexithymia is the result of environmental influences (Gündel et al., 2002), such as traumatization and adversities both in childhood or adulthood. Previous studies have shown that childhood adversities often lead to alexithymia in adulthood, which is due to a lack of introjection of how differentiate emotional states, regulate arousal, and respond to challenge life events (Zlotnick et al., 2001). On the other hand, some psychophysiological evidences showed that alexithymia might also bias the perception of stress and lead to a decoupling between subjective and physiological responses to it (Martin and Pihl, 1986; Näätänen et al., 1999; Stone and Nielson, 2001). Hence, alexithymia might lead to an inaccurate self-perception of stressful events, which might in turn prevent an individual from coping effectively with the stressors and, as consequence, prolong the exposure to stress itself (Martin and Pihl, 1985). The fact that in the present study we have found a more evident prevalence of childhood trauma, current life events, and alexithymic trait in CM+MOH patients with respect to episodic migraineurs suggest the complexity of CM+MOH and that these factors may act together and predispose the development of this complex clinical condition. We hypothesize that early traumatic experiences drive to the development of a kind of psychological vulnerability which—in the case of additional stressful events—sets the ground for chronic evolution of pain. This does not exclude a genetic predisposition to migraine given that these patients do not develop other types of chronic or recurrent pain. The severity of trauma/stressful event might be the medium toward the worst evolution of pain in the direction of chronicity, and alexithymia an additional factor predicting the worst outcome (CM+MOH in our case). Furthermore, the well-known comorbidity with anxiety and mood disorders might be related to such psychological vulnerability, predisposing individuals to ineffective strategies of coping with adverse events. What happens at the neurobiological level in headache patients should be matter of further studies. To date, the pathophysiology of migraine and its chronification is very complex and still needs to be fully understood, being the result of structural and functional alterations (Burstein et al., 2015). Among those causative aspects that could determine the shift from episodic to chronic migraine, we certainly believe that a critical role should be attributed to psychological and psychosocial factors.

A final consideration should be paid to the fact that our two groups of migraneurs differed only in a specific subscale of the TAS-20, that is the one concerning difficulties in identifying feelings and distinguishing between feelings and the bodily sensations of emotional arousal. A recent study has shown that alexithymic individuals could have difficulties not only in identifying their own emotions, but also for what concerns their non-affective interoceptive state (Brewer et al., 2016). For instance, in other fields of research, it has been shown that these persons could be inaccurate in perceiving their own bodily sensations (Herbert et al., 2011) This determines a delay in seeking medical treatment (Carta et al., 2013) and an incorrect assumption of substances that could make them less aware of their body (de Haan et al., 2014). The alexithymic trait in which we found differences, among all those from the TAS-20, seems to be the one mostly corroborating this previous finding about non-affective interoceptive deficits, and that could explain the medication misuse our patients had. However, future studies are needed to further verify this hypothesis.

Our study is not free from limitations. Only CM+MOH patients received a psychiatric evaluation based on DSM-IV criteria (American Psychiatric Association, 1994), while EM were only screened for anxiety and depression using the Hospital Anxiety and Depression Scale (Zigmond and Snaith, 1983). We are aware that co-occurring psychiatric disorders could complicate both headache and medication overuse behaviors. However, we believe that, while the strong comorbid association between depression/anxiety and chronic headache is well-known, it is important to focus on additional psychological components, such as child trauma and recent life events (Galli, 2017), that may explain why some patients with migraine evolve into CM+MOH. We assessed childhood trauma and current stressors using retrospective self-report questionnaires and without addressing the duration of such exposure. However, we feel that these findings provide an important key lecture for a phenomenon that is largely unexplained. Furthermore, this was a cross-sectional study and we are not able to specify the casual trajectories underlying the association among childhood trauma, life events, alexithymia, and medication overuse headache. In this frame, a potential limitation is the lower mean age detected in the EM group as compared to the CM+MOH group, which does not allow to exclude with absolute certainty that some of the patients in the EM group would not evolve into CM+MOH in the next few years. Finally, data collection procedure did not reflect the general migraine population, given that participants were recruited from a neurological clinic. Hence, transferability of these findings to the general practice will require confirmation on larger sub-groups of patients, in multi center studies, and different cultures.

In conclusion, our results showed that childhood trauma, life events, and alexithymia are associated to CM+MOH. From a theoretical point of view, our data add an important element to the identification of the risk factors for the development of this condition. From a practical point of view, they provide useful indications as regards the importance of optimizing patients' management by means of a preliminary thorough evaluation of their psychological and psychosocial history. We believe that patients having experienced childhood traumas, important stressful life events and presenting alexithymic traits should be treated by clinicians also from a psychological point of view due to high risk of negative outcome.

## Author contributions

SB wrote the first draft. CT and FG did revisions. All authors contributed to the planning and development of the study, supervised by CT. All authors read and approved the final manuscript.

### Conflict of interest statement

The authors declare that the research was conducted in the absence of any commercial or financial relationships that could be construed as a potential conflict of interest.
